# Numerical study on the axial collapse of thin-walled columns

**DOI:** 10.1038/s41598-024-56558-5

**Published:** 2024-03-12

**Authors:** Di Wang, Houcheng Fang, Ruilei Xue, Shen Li

**Affiliations:** Department of Mechanical Engineering, Xinjiang University, Ürümqi, China

**Keywords:** Theory and computation, Mechanical engineering

## Abstract

In order to investigate the damage characteristics of structural components under axial compression, thin-walled columns are chosen as a more straightforward construct due to the complex structural properties of composite materials, diverse fiber laying angles, and varied geometries associated with thin-walled columns. Despite the limitations imposed by labor-intensive testing procedures, high costs, and the poor repeatability inherent in experimental research methods, there remains an insufficient exploration of axial compressive damage in columns composed of aluminum and fiber-reinforced polymers. This article utilizes the finite element technique to quantitatively analyze the crushing processes of four materials: aluminum, carbon fiber-reinforced aluminum, carbon fiber-reinforced polymer, and glass fiber-reinforced polymer. It examines the effects of varying fibers and matrix materials on their mechanical attributes. The study also evaluates the impact of different cross-sectional designs on the mechanical behavior of the columns.

## Introduction

A fibre-reinforced composite represents a multi-phase solid resulting from the combination of two or more components possessing distinct properties. Owing to its exceptional mechanical strength and corrosion resistance, this material has found extensive applications in industrial sectors such as aerospace and automotive industries^1,2^. Specifically, fibre-reinforced composites have supplanted ceramics and metals traditionally used in column applications due to their superior bearing and energy absorption properties. Owing to their anisotropic nature, these materials exhibit diverse mechanical properties in different directions, rendering them susceptible to factors such as structural form, dimensions, laying angles, temperature, and laying techniques^3,4^. Additionally, the failure behavior of these composites surpasses the complexity observed in homogeneous isotropic materials, exhibiting distinct patterns of matrix degradation, delamination, and fiber breakage. During the degradation process, multiple types of failures interplay, complicating the destructive behavior^5–7^. The investigation of failure mechanisms in fiber-reinforced composites remains a complex and unresolved issue due to their intricate structures and diverse forms of damage.

Axial crushing serves as a common method for evaluating the energy-absorbing capabilities of fiber-reinforced composite laminates^8–10^. Numerous studies have examined the energy-absorbing properties of composite columns under varying impact velocities. These investigations suggest that by employing a systematic approach to fiber-reinforced composite design and fiber orientation, one can modify the failure mode towards increased durability, thereby enhancing the column's energy-absorption capacity^10–13^. Research indicates that employing a well-designed fiber-reinforced composite structure and fiber orientation method can transform the column body's compression failure process into a more gradual and prolonged sequence, thereby enhancing its energy-absorption capacity.

Cross section is a major feature of the geometry of column. Zhang et al.^14^ studied the influence of geometry, filling type and mixture of various materials on GFRP thin wall column's energy-absorbing performance. It is found that the amount of energy absorbed can be greatly improved when the structure of the structure is modified, and the filler foam and the compound is replaced by metallic material. The cross-section significantly influences a column's geometric properties. Additionally, research indicates that modifying structural parameters and substituting foam fillers and composites with metallic materials can substantially enhance energy absorption capabilities. Li et al. conducted a comparative analysis of the deformation characteristics, deformation patterns, energy-absorbing capabilities, and shock resistance between single-column and double-round columns under axial loads^15^. Their findings revealed that both single-column and double-round columns, when filled with foam, exhibit superior structural strength. Additionally, the aspect ratio emerges as a crucial parameter delineating a column's geometry. In a study by Farokhi et al.^10^, the impact of varying aspect ratios on the axial fracture energy of CFRP columns was explored. The research highlighted that while increasing the aspect ratio results in a marginal rise in energy absorption, it markedly decreases once surpassing a critical length.

Fiber-reinforced composites comprise both fiber and matrix materials, with the performance of each profoundly influencing the composite's mechanical properties. Carbon fiber, renowned for its mechanical strength and corrosion resistance, ranks among the most prevalent synthetic fibers globally. However, its high cost and inherent brittleness present notable limitations^10^. Glass fibre reinforced composite is extensively applied in mechanical, chemical, and transport because of its light-weight, corrosion-resistant, and insulating performance. Literature^16^ has investigated the variation of the fracture course and the damage parameters of the CFRP and GFRP columns subjected to the axial stress. The results show that CFRP and GFRP can reduce their maximum stress and strain when they are compressed because of the change in fibre performance and the impact surface. In their study, Sun et al. conducted a comparative analysis of GFRP and CFRP thin-walled circular columns, evaluating their impact resistance under radial loads. Their research confirmed that while CFRP exhibits superior mechanical properties, it demonstrates marginally inferior energy absorption and buffering capabilities compared to GFRP^17,18^. Additionally, research indicates that incorporating two or more fibers as reinforcement enhances the properties of FRP^8^. Research indicates that incorporating two or more fibers as reinforcement enhances the properties of FRP. Additionally, scholars have leveraged the benefits of both GFRP and CFRP in hybrid structures to enhance material properties against impact loads. Building upon this approach, several studies have utilized hybrid fiber composites to fabricate thin-walled glass steel columns, assessing the impact resistance across various geometries and sections^19^.

The matrix material in fiber-reinforced composite materials commonly comprises resin or metal. Notable metal matrix materials encompass aluminum, copper, lead, magnesium, nickel, silver, and titanium. Shashi provided an extensive overview of the classification, manufacturing processes, and mechanical properties of FRPs^20^. Research suggests that metal matrix fiber-reinforced composites exhibit superior wear resistance, specific strength, and thermal stability^21–23^. Nonetheless, further studies are necessary to ascertain their energy-absorbing capabilities.

The fiber layup angle significantly influences the performance of FRP columns. Research indicates that cross-laid CFRP columns exhibit progressive deformation or failure. Specifically, a greater fiber layup angle correlates with an increased fracture load efficiency^24^. To delve deeper into the impact of fiber layers on deformation, damage, and energy absorption, Sun et al. performed experiments on composite column bodies with varying layup angles (25°, 50°, 75°, and 90°) during the axial collapse process^17,18^. Similarly, Xie et al. employed both experimental and simulation approaches to investigate how different fiber layup angles affect the axial crushing energy absorption properties of thin-walled circular columns^25,26^. The study concludes that fiber orientation significantly influences macro damage, with the energy-absorption rate initially increasing and subsequently decreasing as the fiber layup angle varies. Optimal efficiency is observed at a fiber angle of ± 45°, while employing a 0° or 90° layup orientation enhances the material's energy absorption during failure.

The finite element method was systematically employed to investigate the mechanical properties of fiber-reinforced materials under axial compressive loads within the realm of composite laminate theory. The pivotal point for progressive degradation of the column body is the maximum load, closely linked to structural strength. Consequently, this article adopts the maximum load as a benchmark. The paper is organized as follows: Section “Methods and models” delves into methods and models, encompassing anisotropic material definitions, failure mechanisms, and detailed analyses of aluminum and GFRP column crushing models. Section “Numerical examples and discussions” presents simulations and discussions, covering collapse procedures and insights derived from Al columns, CF/Al columns, CFRP, and GFRP columns. The concluding remarks are provided in Section “Conclusions”.

## Methods and models

This section aims to validate and assess the material constitutive equation, damage model, and collapse failure mechanism for thin-walled columns. The aforementioned models and techniques form the basis for all numerical computations conducted in this study.

### Anisotropic material constitutive equation

**Figure 1:**
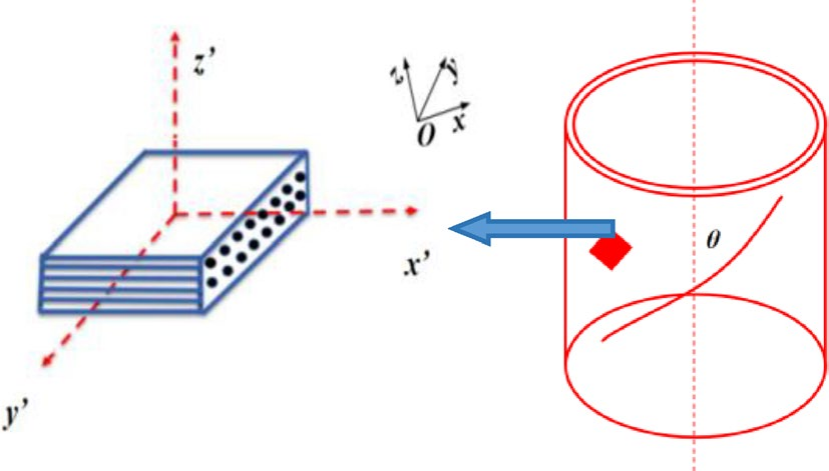
Spatial structure diagram of an anisotropic material.

Fiber-reinforced composite materials exhibit typical anisotropic behavior, implying variations in mechanical properties across different directions. For such anisotropic materials, Green's strain is determined using a transformation matrix, followed by the calculation of a second-order Piola–Kirchhoff stress. The Piola–Kirchhoff stress is transformed into Green's stress, and subsequently, the nodal force is determined. This study employs the full Lagrange formulation, obviating the need to compute the J strain rate. It's important to note that the Young's modulus, shear modulus, and Poisson's ratio exhibit directional variations within the anisotropic material. Typically, the parametric procedure for the material encompasses nine distinct parameters. Within this figure, x–y–z denotes the global coordinate system, while x′–y′–z′ signifies the material coordinate system. Assuming that the cosine values of the angles between each axis of the material coordinate system and the global coordinate system are represented as *l*_*i*_, *m*_*i*_ and *n*_*i*_ for *i* = 1, 2, and 3 respectively, the Piola–Kirchhoff stresses *S* can be determined from Green's strain *ε*. Furthermore, a specific spatial relationship among the materials is depicted in Fig. 1.

1 $$ S = C \cdot \varepsilon = T^{t} C_{l} T \cdot \varepsilon $$

In Eq. (1), the transformation matrix *T* and the symmetrical constitutive matrix *C*_*l*_ can be expressed as:

2 $$ T = \left[ {\begin{array}{*{20}c} {l_{1}^{2} } & {m_{1}^{2} } & {n_{1}^{2} } & {l_{1} m_{1} } & {l_{1} m_{1} } & {n_{1} l_{1} } \\ {l_{2}^{2} } & {m_{2}^{2} } & {n_{2}^{2} } & {l_{2} m_{2} } & {m_{2} n_{2} } & {n_{2} l_{2} } \\ {l_{3}^{2} } & {m_{3}^{2} } & {n_{3}^{2} } & {l_{3} m_{3} } & {m_{3} n_{3} } & {n_{3} l_{3} } \\ {2l_{1} l_{2} } & {2m_{1} m_{2} } & {2n_{1} n_{2} } & {(l_{1} m_{2} + l_{2} m_{1} )} & {(m_{1} n_{2} + m_{2} n_{1} )} & {(n_{1} l_{2} + n_{2} l_{1} )} \\ {2l_{2} l_{3} } & {2m_{2} m_{3} } & {2n_{2} n_{3} } & {(l_{2} m_{3} + l_{3} m_{2} )} & {(m_{2} n_{3} + m_{3} n_{2} )} & {(n_{2} l_{3} + n_{3} l_{2} )} \\ {2l_{3} l_{1} } & {2m_{3} m_{1} } & {2n_{3} n_{1} } & {(l_{3} m_{1} + l_{1} m_{3} )} & {(m_{3} n_{1} + m_{1} n_{3} )} & {(n_{3} l_{1} + n_{1} l_{3} )} \\ \end{array} } \right] $$

3 $$ C_{l}^{ - 1} = \left[ {\begin{array}{*{20}c} {\frac{1}{{E_{11} }}} & { - \frac{{\nu_{21} }}{{E_{22} }}} & {\frac{{\nu_{31} }}{{E_{33} }}} & 0 & 0 & 0 \\ { - \frac{{\nu_{12} }}{{E_{11} }}} & { - \frac{{\nu_{12} }}{{E_{11} }}} & { - \frac{{\nu_{12} }}{{E_{11} }}} & 0 & 0 & 0 \\ { - \frac{{\nu_{13} }}{{E_{11} }}} & { - \frac{{\nu_{23} }}{{E_{22} }}} & {\frac{1}{{E_{33} }}} & 0 & 0 & 0 \\ 0 & 0 & 0 & {\frac{1}{{G_{12} }}} & 0 & 0 \\ 0 & 0 & 0 & 0 & {\frac{1}{{G_{23} }}} & 0 \\ 0 & 0 & 0 & 0 & 0 & {\frac{1}{{G_{31} }}} \\ \end{array} } \right] $$

Note that Eqs. (1)–(3) are all from^27^.

### Continuous damage model for composite

This study delineates the mechanical characteristics of fiber-reinforced materials through a macro-phenomenological approach. This methodology treats the composite's fiber and matrix as a singular entity, thereby disregarding their individual distinctions and rendering the thin-wall structure homogeneous and continuous. The collective mechanical behavior of a single layer provides insights into its overall rigidity, strength, and other inherent properties. Findings indicate the viability of this method for evaluating both single-layer and multi-layer materials, facilitating investigations into their geometric, distributional, and mechanical attributes as well as their interrelationships. Experimental analyses have enabled the determination of macroscopic material properties, such as elasticity factor and strength.

The failure criterion serves as an essential foundation for assessing strength and forecasting material damage in fiber-reinforced composites. Over time, researchers have persistently endeavored to refine the strength criteria for composite materials. Currently, numerous theoretical models exist to enhance the precision of predicting failure behaviors in composite structures. Given the pronounced nonlinear damage in the shear directions *G*_12_ and *G*_13_ of anisotropic materials, contemporary failure criteria for fiber-reinforced composites emphasize the materials' nonlinear shear behavior, enabling more effective prediction and control of failure occurrences. The Chang-Chang failure criterion, introduced by Chang^28^ and subsequently endorsed by various scholars^29–31^, elucidates the nonlinear shear characteristics of fiber-reinforced anisotropic materials. According to this criterion, stress-induced damage may occur in composite materials. Thus, by integrating the failure criterion with the stress state of composites, the formulation for the Chang-Chang failure criterion is as follows:

When the fibre produces tensile failure, *σ*_11_ > 0

4 $$ \left( {\frac{{\sigma_{11} }}{{X_{t} }}} \right)^{2} + \beta \left( {\frac{{\sigma_{12} }}{{S_{c} }}} \right) \ge 1 $$

When the fibre produces compression failure, *σ*_11_ < 0

5 $$ \left( {\frac{{\sigma_{11} }}{{X_{c} }}} \right)^{2} + \beta \left( {\frac{{\sigma_{12} }}{{S_{c} }}} \right) \ge 1 $$

When the matrix has tensile failure, *σ*_22_ > 0

6 $$ \left( {\frac{{\sigma_{22} }}{{Y_{t} }}} \right)^{2} + \beta \left( {\frac{{\sigma_{12} }}{{S_{c} }}} \right) \ge 1 $$

When the matrix has compression failure, *σ*_22_ < 0

7 $$ \left( {\frac{{\sigma_{22} }}{{2S_{c} }}} \right)^{2} + \left[ {\left( {\frac{{Y_{e} }}{{2S_{c} }}} \right)^{2} - 1} \right]\frac{{\sigma_{22} }}{{Y_{e} }} + \left( {\frac{{\sigma_{12} }}{{S_{c} }}} \right)^{2} \ge 1 $$

In Eqs. (4)–(7), the radial shear strength is represented by *S*_*c*_*. X*_*t*_, *X*_*c*_, *Y*_*t*_ and *Y*_*c*_ are the strength parameters for tension and compression in the *x* and *y* directions respectively.

The Chang-Chang failure criterion has consistently proven to be a precise and efficient approach for analyzing failures in composite materials, as evidenced by multiple studies. Specifically, an integral across the thickness direction within the shell element was utilized to ascertain the contribution of thickness deformation to nodal forces. This selected shell element is particularly suitable for thin-shell structures, effectively capturing the structure's thickness in a unified direction.

**Figure 2:**
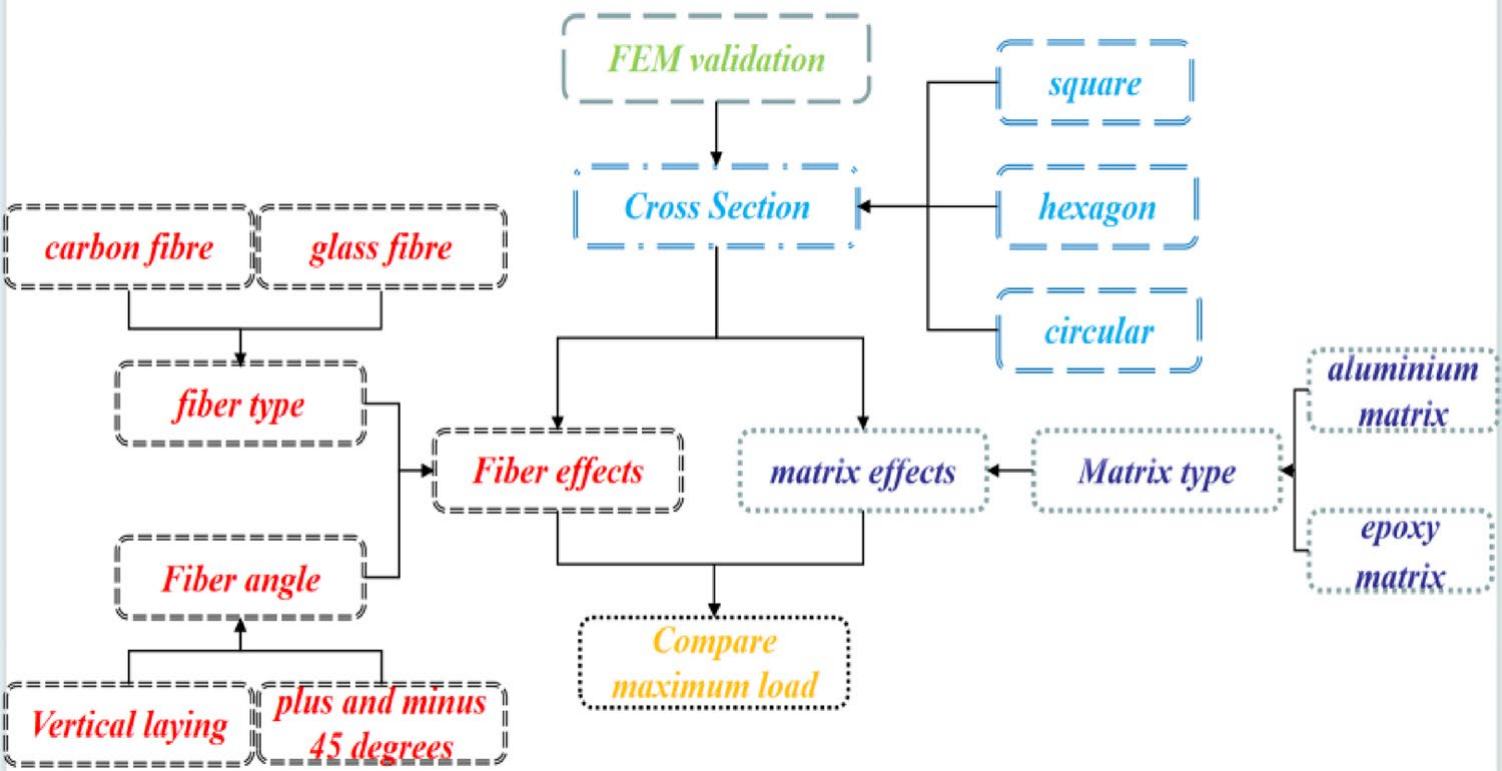
Technical routes.

This study utilizes the LS-DYNA software for finite element simulations. The methodology of this research is illustrated in Fig. 2.

### Model and validate for aluminum column

**Figure 3:**
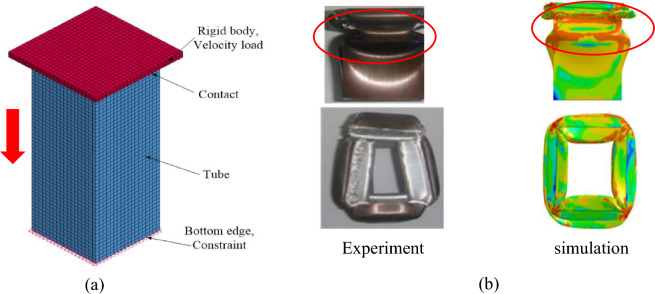
Finite element model of aluminum column collapse. (**a**) Finite element model of Al column. (**b**) Failure pattern of Al column^32^.

To investigate the influence of fiber on the mechanical properties of composite columns, we examine the crushing behavior of Al columns with and without fiber reinforcement. Without fiber, Al columns exhibit uniformity, isotropy, as well as both elastic and plastic behaviors. Based on the square Al column crushing experiment^32^, we establish a thin-walled Al column model, depicted in Fig. 3a.

The column features a wall thickness of 1.2 mm, a diameter of 38 mm, and a height of 95 mm. Given its elastic–plastic behavior, we characterize the mechanical properties of Al using an elastic–plastic constitutive equation. The column is modeled using 4-node shell elements. The initial mesh size of the tube is 2 mm (validated through a convergence analysis). The material properties for Al are specified as follows: Young's modulus *E* = 69 GPa, mass density *ρ* = 2700kg/m^3^, yield strength *σ*_*s*_ = 90 MPa, tangent modulus *E*_*T*_ = 2 GPa, and Poisson's ratio *υ* = 0.33^33,34^. In this study, we adopt *MAT_POWER_LAW_PLASTICITY (MAT18) to represent aluminum's behavior. The rigid wall is modeled using solid elements, treating it as a rigid body without considering damage, and is defined using the *MAT model _RIGID (MAT20). Within the Finite Element Method (FEM), the rigid wall is discretized, ensuring it remains rigid without distortions or damages during loading. A node cluster forms at the lower end of the compressed column, constrained entirely. With an imposed speed of 0.1 m/s on the rigid wall, the maximum axial load-bearing distance is determined as 47.5 mm. Employing a surface-to-surface algorithm, we compute the contact force between the rigid wall and the Al column. Figure 3b illustrates the post-crushing damage pattern of the specimen, highlighting pronounced plastic deformation, particularly in the top section of the thin-walled Al column. Conversely, the column's bottom section retains its initial shape predominantly. The simulation and experimental results exhibit a high degree of congruence in terms of deformation and failure modes.

**Figure 4:**
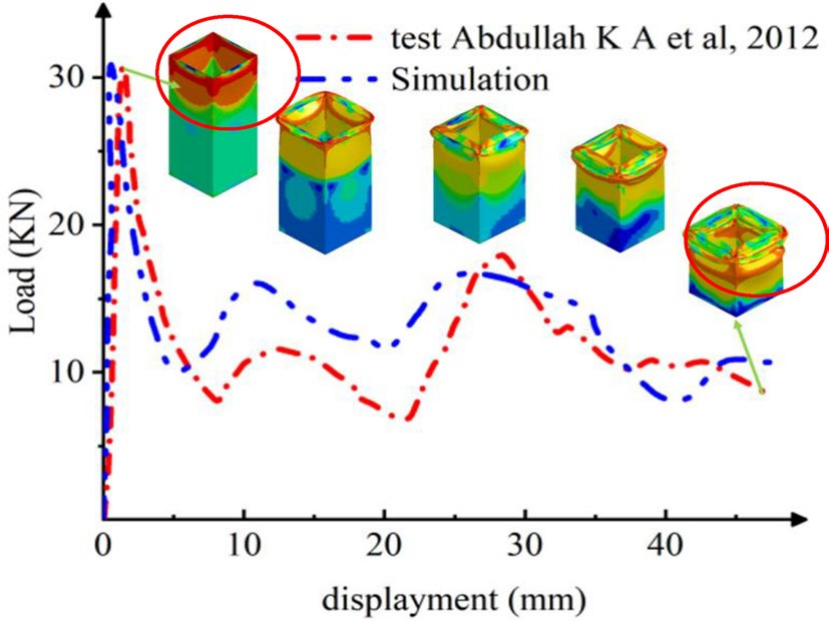
Crushing process of Al column.

Figure 4 presents a load–displacement profile, highlighting a close correlation between experimental and simulated data. Both datasets indicate peak load values of 31.14 kN for the experimental trial and 31.7 kN for the simulation, reflecting a minor discrepancy of approximately 1.8%. This alignment is evident in Fig. 4. As the Al column experiences axial compression, the load initially rises sharply before stabilizing into an oscillatory pattern. A linear relationship emerges initially, suggesting elastic deformation without internal damage. However, prolonged compression causes specific sections of the aluminum column to undergo crushing failure, substantially reducing its load-bearing capacity—indicating a subsequent phase. Remarkably, the Al column maintains its load-carrying capacity towards the end of its lifespan, resulting in a consistent average load over a specified duration. Local buckling failure mode results in a diminished overall load-bearing capacity within the structure, manifesting in fluctuations of the load-bearing capacity corresponding to displacement.

### Model and validate for composite column

**Figure 5:**
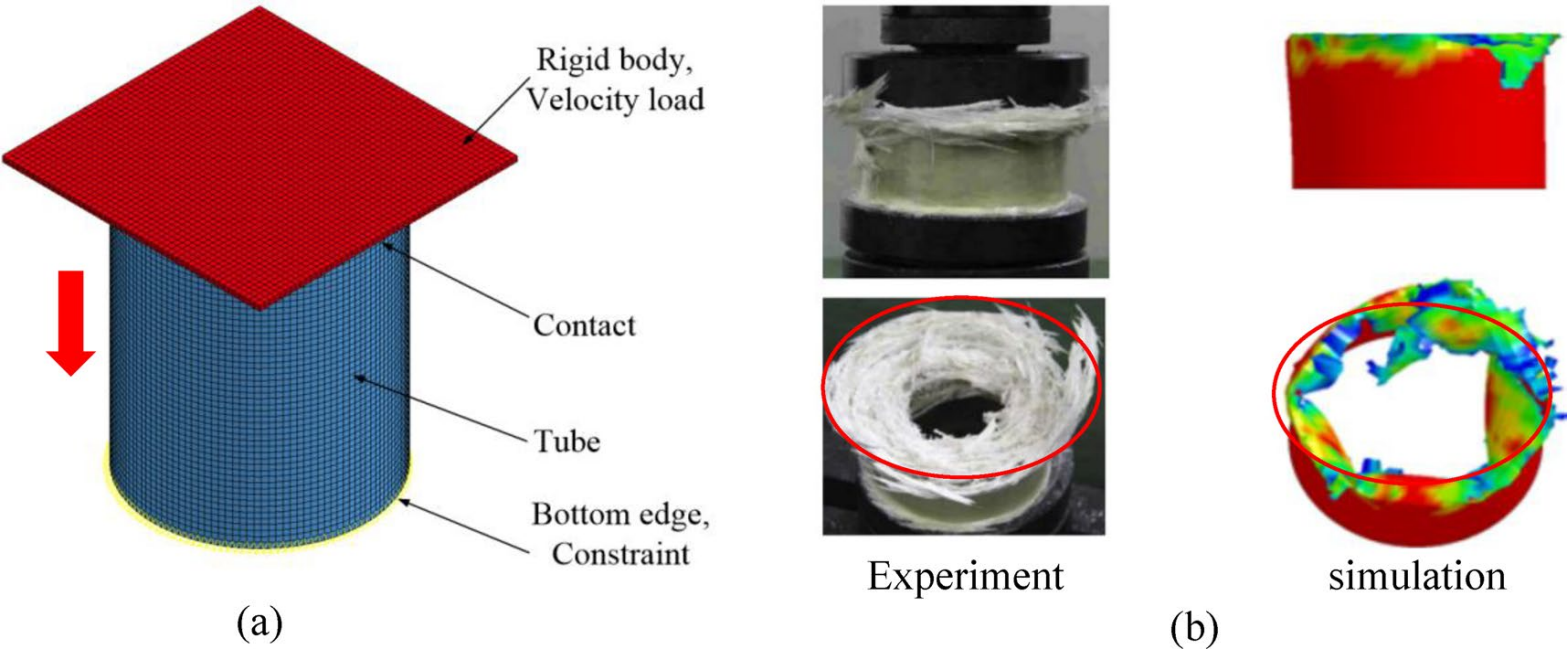
Finite element model of composite column collapse. (**a**) Finite element model of composite column. (**b**) Failure pattern of composite column^14^.

In analyzing the axial crush behavior of composite column bodies through simulation, computational efficiency often decreases due to the intricate nature of multi-layer shell elements, leading to a significant computational burden. To ensure both accuracy and efficiency, we adopt a single-layer shell approach to model the axial fracture in thin-walled composite structures. This streamlined methodology simplifies the composite material representation, offering computational precision while mitigating issues related to mesh size variations seen in multi-layered models. Such an approach has been widely recognized for its efficacy in axial crush simulations^14^. Accordingly, this section employs the single-layer shell element technique to simulate the collapse of thin-walled composite structures. Drawing upon insights from^14^, we developed a finite element model for a GFRP column with a circular cross-section, characterized by dimensions of 100 mm in height, an 80 mm inner diameter, and a 2.4 mm thickness, as illustrated in Fig. 5a. The composite features fibers oriented at alternating ± 75° angles. The initial mesh size of the tube is 2 mm (validated through a convergence analysis). Representing the rigid wall, solid elements are employed and treated as rigid bodies. The composite's orthotropic behavior is governed by the MAT54 progressive damage model, anchored in the Chang-Chang failure criterion. Detailed material parameters are provided in Table 1. Similar to the Al column model, a node set is constrained at the column's base, while the rigid wall moves at a speed of 0.1 m/s. Employing a surface-to-surface contact algorithm, we compute the contact force between the rigid wall and the column, addressing potential self-contact issues post-deformation via a singular surface contact algorithm.

**Table 1:** Material properties of GFRP^14^.

Parameter	Settings
Modulus in longitudinal direction, *E*_1_	37.9 GPa
Modulus in transverse direction, *E*_2_ = *E*_3_	11.5 GPa
Major Poisson’s ratio, *υ*_12_ = *υ*_13_	0.29
Minor Poisson’s ratio, *υ*_23_	0.088
Major Shear modulus, *G*_12_ = *G*_13_	4.5 GPa
*S*_*b*_Minor Shear modulus, *G*_23_	3.8 GPa

Figure 5b illustrates the comparison between simulation and experimental failure patterns for the GFRP column. The figure reveals multiple cracks predominantly located in the column's upper portion. In contrast, the bottom section exhibits minimal distortion, with the central hole closely resembling its original form. Additionally, both frontal and vertical perspectives align closely with the experimental data regarding deformation and failure modes.

**Figure 6:**
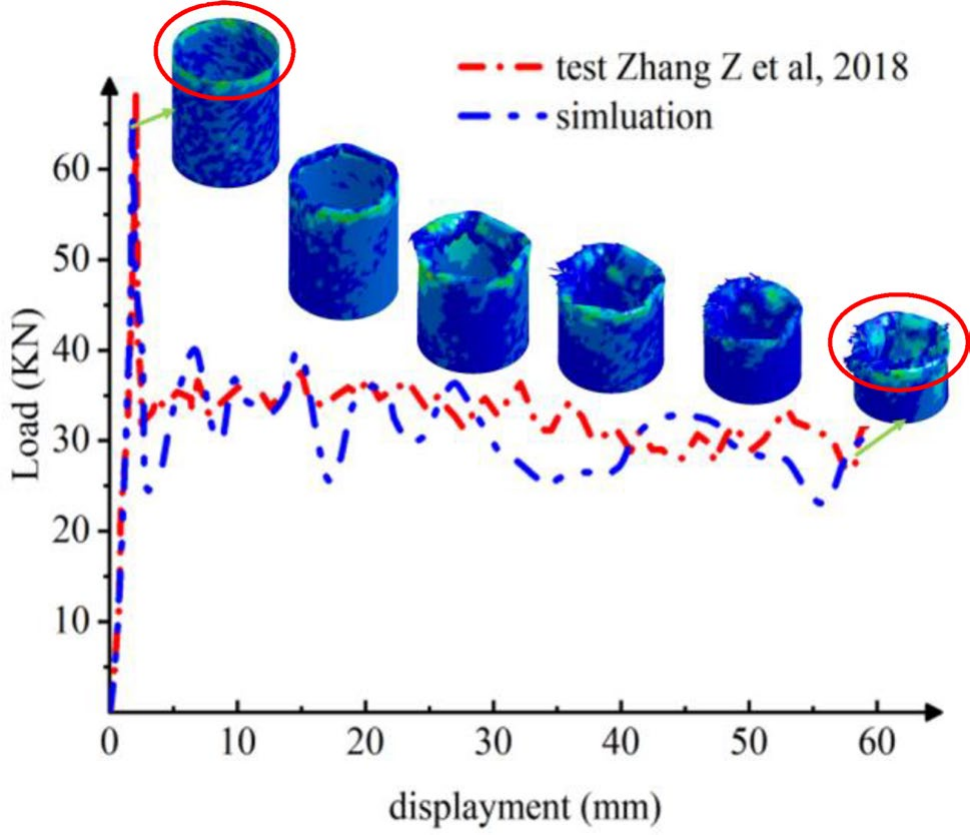
Crushing process of composite column^32^.

Figure 6 presents the load–displacement curve detailing the crushing process for GFRP, exhibiting similarities to the behavior observed in Al columns. Specifically, in the GFRP scenario, the bearing force against the rigid axial wall initially rises sharply, subsequently dropping to 35 KN at a faster rate before stabilizing at a lower magnitude. This behavior predominantly results from rupture-induced failures, leading to a diminished overall carrying capacity. In contrast, Al columns typically experience yield failures, characterized by irreversible significant deformations. As a result, Al columns sustain their load longer during the secondary crushing phase compared to GFRP columns, which exhibit a shorter lifespan with reduced fluctuations. The peak load observed in testing reaches approximately 70 KN, closely aligned with the simulated maximum load of 66 KN, translating to a minimal error margin of merely 5.7%, as depicted in Fig. 6. This close correlation underscores the efficacy and accuracy of the modeling approach employed. Upon the onset of local buckling, the load-bearing capacity of the composite material experiences a swift reduction. Subsequently, the column body undergoes a progressive sequence of crushing and tearing failures initiated by the initial local buckling instability.

## Numerical examples and discussions

In this section, we focus on examining volume variables while evaluating the impact of cross-sectional shapes on fracture properties. Specifically, we compare three commonly utilized column cross-sections in engineering: normal quadrangle, regular hexagonal, and circular shapes. To maintain consistency, the geometry of composite column bodies with varying fibers and matrices remains equivalent to that of metallic aluminum columns. Concurrently, this study delves into the structural implications of internal fibers by contrasting aluminum columns with Cf/Al. We also analyze how the matrix material influences structural behavior, drawing comparisons between Cf/Al and CFRP. Additionally, our findings elucidate how fiber material types, particularly when comparing with GFRP, significantly affect structural mechanical performance. We further investigate the influence of fiber laying angles on these properties. The research encompasses four groups, totaling 21 cases as delineated in Table 2. Each group features three distinct cross-sections: rectangular, hexagonal, and circular, all sharing dimensions of 152 mm circumference, 95 mm height, and 1.2 mm thickness. For materials like Cf/Al, CFRP, and GFRP, calculations consider two laying orientations: [0°]_10_ (vertical) and [± 45°]_5_ (45° cross-laying).

**Table 2:** Details of the twenty-one numerical cases.

	Al	Cf/Al		CFRP		GFRP	
		[0°]_10_	[$\pm$ 45°]_5_	[0°]_10_	[$\pm$ 45°]_5_	[0°]_10_	[$\pm$ 45°]_5_
Square	Case 1	Case 4	Case 7	Case 10	Case 13	Case 16	Case 19
Hexagonal	Case 2	Case 5	Case 8	Case 11	Case 14	Case 17	Case 20
Circular	Case 3	Case 6	Case 9	Case 12	Case 15	Case 18	Case 21

A comprehensive constraint is applied to the column's lower nodes, subjected to a 0.1 m/s load on the solid wall nodes. All axial compression distances are set at 47.5 mm. Contact interactions between the column and rigid wall, including self-contact scenarios, are managed using surface-to-surface and single surface contact algorithms. The anisotropic constitutive equation characterizes the behavior of fiber-reinforced composites, while the Chang-Chang failure criterion and continuous damage model depict their failure behaviors. In this study, Crush Force Efficiency (CFE) serves as a pivotal index to evaluate axial damage in thin-walled tubular bodies for simulation analyses. The CFE is defined as the ratio between the average force and the maximum peak force. The mathematical representation of CFE is delineated in Eq. (8).

8 $$ {\text{CFE}} = \frac{{F_{{{\text{ave}}}} }}{{{\text{GPCF}}}} \times 100\% $$

The Global Peak Crush Force (GPCF) represents the highest peak force encountered throughout the entire deformation process. During the axial crush test of CFRP thin-walled cylindrical columns, this maximum peak force is typically observed during the compaction phase under the upper platen. Meanwhile, the Average Force $\left( {F_{{{\text{ave}}}} } \right)$ quantifies the energy absorbed per unit length during the collision, offering insights into the overall energy absorption characteristics of the structures. This calculation is defined by Eq. (9).

9 $$ F_{{{\text{ave}}}} = \frac{1}{d}\int_{0}^{d} F \left( x \right){\text{d}}x $$

### Crushing process of Al column with an axial loading

**Figure 7:**
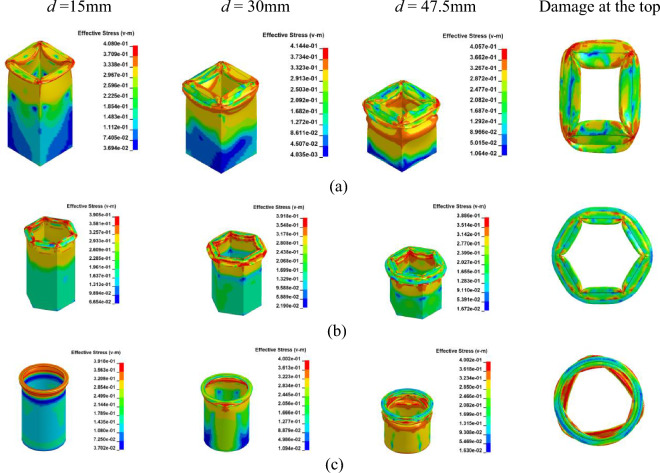
Simulation of the Al columns crushing process. (**a**) Square section, Case 1. (**b**) Hexagonal section, Case 2. (**c**) Circular section, Case 3.

**Figure 8:**
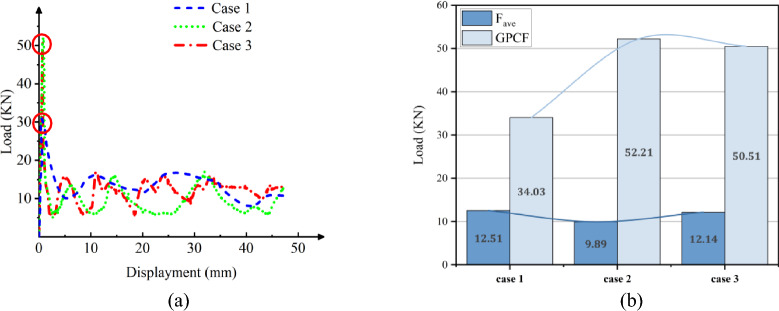
The load–displacement curve of Al columns during the crushing process. (**a**) Load and displacement curve for case 1–3. (**b**) Comparison for case 1–3.

Utilizing the same physics parameters outlined in Section “Model and validate for aluminum column” and employing an adaptive mesh method, we have examined the axial fracture and failure mechanisms of Al columns to enhance computational accuracy. Figure 7 illustrates that, across the three distinct sectional shapes, the pronounced plastic deformation results in consistent layered folds primarily at the column's upper portion. Conversely, the bottom section remains unaltered and retains its original configuration when viewed vertically. As depicted in Fig. 8a, the load–displacement curves for Al columns with varying section shapes exhibit striking similarities. Specifically, in Fig. 8b, the hexagonal section demonstrates a marginally higher maximum load of 52.21 KN compared to the circular counterpart at 50.51 KN. These findings indicate comparable structural integrity and compressive resistance across the shapes, albeit superior to that of rectangular columns.

The CFE values for Cases 1–3 are 37%, 19%, and 24%, respectively. Regarding aluminum tubes, the crushing force efficiency proves superior for a rectangular cross-section in comparison to circular and square cross-sections. This observation suggests that a smaller fluctuation of load force during the crushing process corresponds to a more stable crushing process.

### Crushing process of Cf/Al column with an axial loading

Cf/Al is extensively utilized in aviation due to its exceptional integrated properties, including high specific strength, modulus, thermal and electrical conductivity, elevated temperature resilience, wear resistance, and a minimal coefficient of thermal expansion. In this study, we aim to numerically analyze the axial crushing behavior of Cf/Al columns and assess their outstanding mechanical performance.

**Figure 9:**
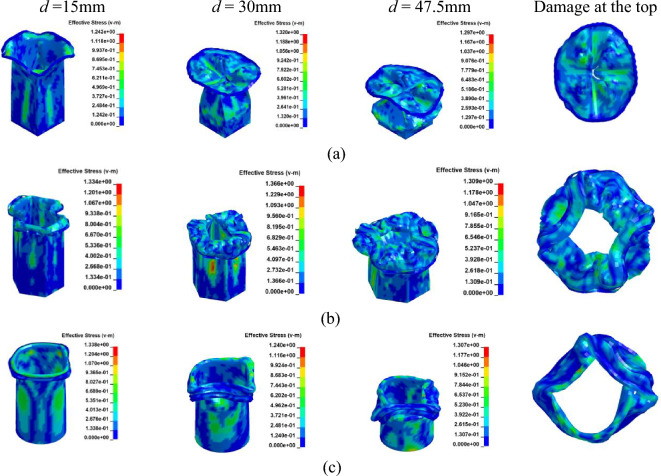
Simulation of the Cf/Al column crushing process, at [0°]_10_. (**a**) Square section, Case 4. (**b**) Hexagonal section, Case 5. (**c**) Circular section, Case 6.

**Figure 10:**
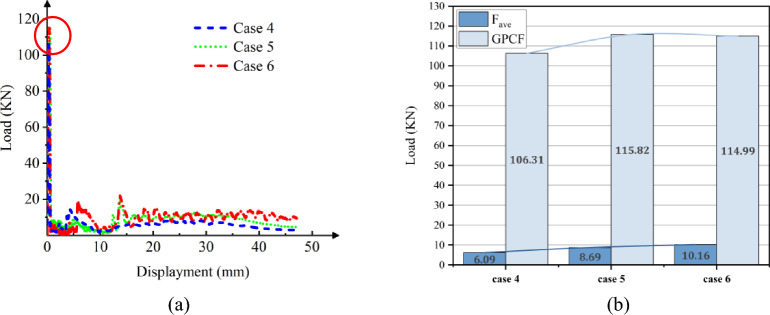
The load–displacement curve of Cf/Al columns during crushing, at [0°]_10_. (**a**) Load and displacement curve for case 4–6. (**b**) Comparison for case 4–6.

**Figure 11:**
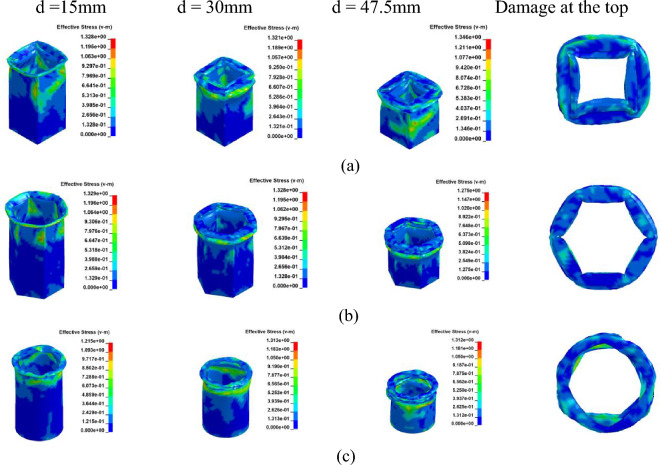
Simulation of the Cf/Al columns crushing process, at [± 45°]_5_. (**a**) Square section, Case 7. (**b**) Hexagonal section, Case 8. (**c**) Circular section, Case 9.

**Figure 12:**
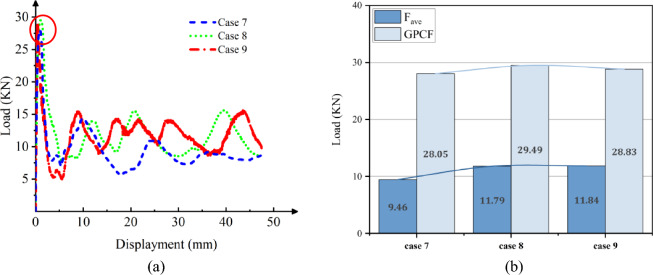
The load–displacement curve of Cf/Al columns during crushing, at [± 45°]_5_. (**a**) Load and displacement curve for case 7–9. (**b**) Comparison for case 7–9.

Accordingly, Finite Element (FE) models corresponding to Case 4 through Case 9, as listed in Table 2, incorporate various laying angles and cross-sectional configurations. The material properties of the Cf/Al column models are sourced from references^35,36^, as detailed in Table 3. Figure 9 depicts the damage progression during axial collapse across various sectional configurations at a [0°]_10_ orientation. The relationship between load and displacement is presented in Fig. 10. Meanwhile, Figs. 11 and 12 illustrate the crushing dynamics for a [± 45°]_5_ orientation.

**Table 3:** Material properties of Cf/Al^35,36^.

Parameter	Settings
Modulus in longitudinal direction, *E*_1_	302.7 GPa
Modulus in transverse direction, *E*_2_ = *E*_3_	23.5 GPa
Major Poisson’s ratio, *υ*_12_ = *υ*_13_	0.28
Minor Poisson’s ratio, *υ*_23_	0.41
Major Shear modulus, *G*_12_ = *G*_13_	14.7 GPa
Minor Shear modulus, *G*_23_	10 GPa

As illustrated in Figs. 9 and 11, owing to the tube's matrix material being aluminum, the tube wall predominantly adheres to the damage pattern of aluminum tubes during the process of crushing failure, resulting in the formation of numerous crumpled and stacked folds.

As observed from Figs. 10a and 12a, during the initial phase of crushing, there's a proportional increase between the load and displacement, termed as the elasticity stage. This relationship is influenced by the Young's modulus. Notably, when fibers are vertically oriented, the load escalates more rapidly compared to a ± 45° orientation. This is attributed to the fibers being orthogonal to the cross-section, enhancing the Young's modulus along the fiber direction and consequently bolstering the member's axial strength.

As depicted in Figs. 10b and 12b, during the crushing process, the vertical fiber orientation exhibits a higher peak than the ± 45° orientation. This difference arises because the principal stress reaches its maximum when oriented at ± 45° perpendicular to the shaft. Consequently, the brittle material tends to fail along this ± 45° direction. Placing fibers at a ± 45° angle diminishes their reinforcing effect, resulting in a reduced peak load. The CFE values of Case 4–6 were 6%, 8% and 9%, respectively, and those of Case 7–9 were 34%, 40%, and 41%, respectively. When the fibers were laid at 45°under the same cross-section conditions of collapse, the fluctuation of the load force was smaller, and the collapse process was more stable, and the fibers and the matrix still retained a certain had-in capacity after fracture.

Given the high ductility and plasticity of aluminum (Al), its failure predominantly arises from pronounced yield distortions, resulting in top creasing. However, the presence of fibers in Cf/Al columns mitigates rupture damage along the fiber direction.

From a macroscopic perspective, the column's failure can be attributed to the progressive degradation of the fibers coupled with the inherent plasticity of the metallic material^37^. Notably, the damage pattern of Cf/Al thin-wall columns, characterized by minimal cracks and numerous folds, mirrors that of Al columns, aligning with findings in^38^. With carbon fiber density at 1800 kg/m^3^ and Al at 2700 kg/m^3^, the fiber content in Cf/Al ranges between 30 and 50%^35,36^. Consequently, Cf/Al columns achieve a 10% to 17% weight reduction compared to conventional Al, making them preferred choices in the aviation and automotive industries due to their enhanced structural integrity and specific strength.

### Crushing process of CFRP column with an axial loading

**Figure 13:**
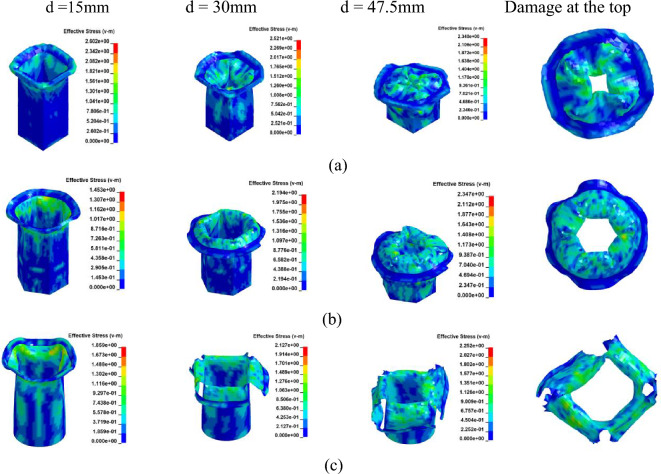
Simulation of the CFRP columns crushing process, at [0°]_10_. (**a**) Square section, Case 10. (**b**) Hexagonal section, Case 12. (**c**) Circular section, Case 13.

**Figure 14:**
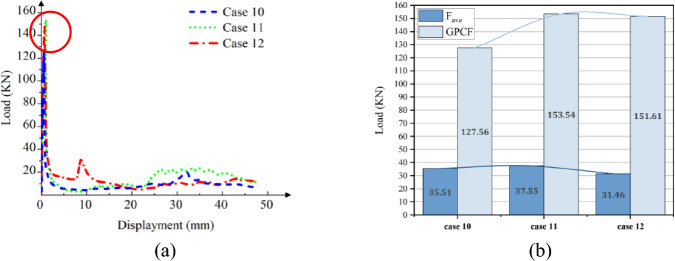
The load–displacement curve of CFRP columns during crushing, at [0°]_10_. (**a**) Load and displacement curve for case 10–12. (**b**) Comparison for case 10–12.

**Figure 15:**
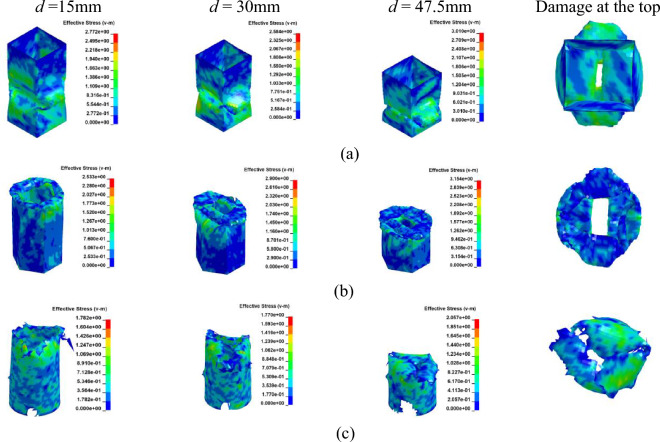
Simulation of the CFRP columns crushing process, at [± 45°]_5_. (**a**) Square section, Case 13. (**b**) Hexagonal section, Case 14. (**c**) Circular section, Case 15.

**Figure 16:**
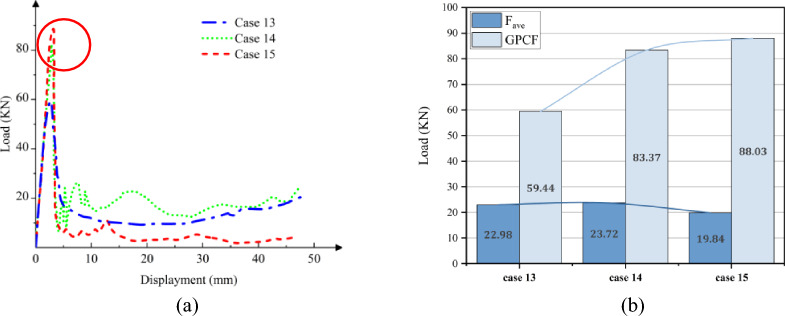
The load–displacement curve of CFRP columns during crushing, at [± 45°]_5_. (**a**) Load and displacement curve for case 13–15. (**b**) Comparison for case 13–15.

The crushing behavior of CFRP columns was analyzed to assess their mechanical properties. It is noteworthy that resin-based carbon fiber composites differ from their aluminum-based counterparts due to distinct substrate selections. Specifically, fiber-reinforced composite materials exhibit significant variations in mechanical performance attributes such as Young's modulus, cross-section contraction factor, and a marginally elevated Poisson's ratio. These disparities inherently influence the structural strength of the columns. Relevant physical properties of the CFRP considered in this study are detailed in Table 4. Consistent with Table 2, models corresponding to Cases 10 through 15 were established. Figure 13 and 14a depict the simulation outcomes for [0°]_10_, while Fig. 15 and 16a illustrate the results for [± 45°]_5_.

**Table 4:** Material properties of CFRP^39^.

Parameter	Settings
Modulus in longitudinal direction, *E*_1_	139 GPa
Modulus in transverse direction, *E*_2_ = *E*_3_	10 GPa
Major Poisson’s ratio, *υ*_12_ = *υ*_13_	0.26
Minor Poisson’s ratio, *υ*_23_	0.4
Major Shear modulus, *G*_12_ = *G*_13_	4.5 GPa
Minor Shear modulus, *G*_23_	3.8 GPa

In the case of a [0°]_10_ layup for CFRP columns, the column's rim exhibits an outward tilt, resembling a blooming effect. As depicted in Fig. 14b and Fig. 16b, the load–displacement curve reveals marginal variations in peak loads between round and hexagonal cross-sections, both surpassing that of the rectangular section. Furthermore, the alternating ± 45° layup showcases behavior akin to the vertical layup. The CFE values for Case 10–12 were 28%, 25%, and 20%, respectively, while those for Case 13–15 were 38%, 28%, and 23%, respectively. In comparison to the vertical laying of fibers, when the fibers are oriented at a ± 45° angle, a smaller fluctuation of the loading force corresponds to a more stable compacting process and higher efficiency.

Due to its slower lifting speed, the material exhibits superior damping and energy-absorbing capabilities compared to Cf/Al columns during laying. The crushing process induces more pronounced damage and cracks in both the cross-section and column wall at [± 45°]_5_, resulting in a lower peak load than that observed at [0°]_10_. When carbon fiber serves as the reinforcing material, the mechanical properties of resin-based composite structures surpass those of Al-based counterparts^40^. Reference^41^ suggests that the fiber does not enhance the shear strength of Cf/Al. Furthermore, owing to its intensified damage, CFRP experiences higher peak stress during its deformation compared to Cf/Al.

### Crushing process of GFRP column with an axial loading

**Figure 17:**
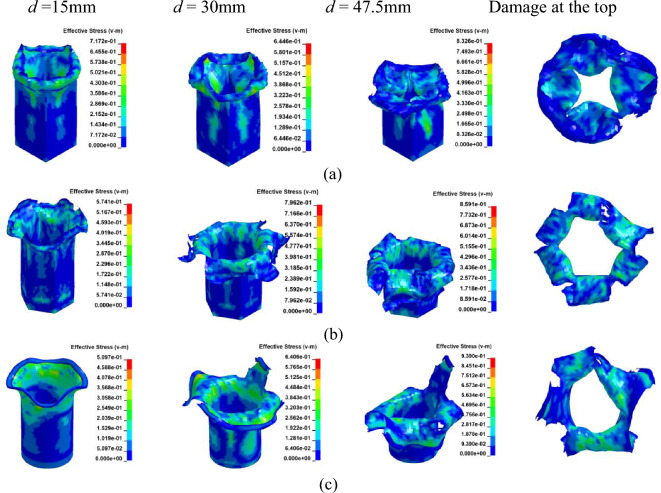
Simulation of the GFRP columns crushing process, at [0°]_10_. (**a**) Square section, Case 16. (**b**) Hexagonal section, Case 17. (**c**) Circular section, Case 18.

**Figure 18:**
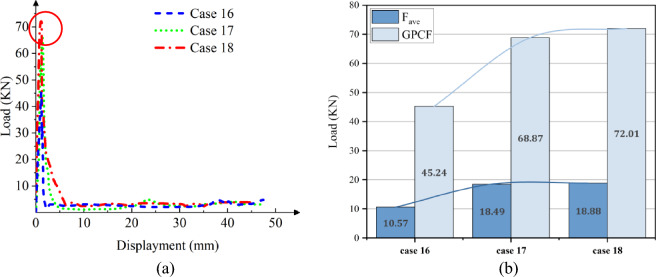
The load–displacement curve of GFRP columns during crushing, at [0°]_10_. (**a**) Load and displacement curve for case 16–18. (**b**) Comparison for case 16–18.

While CFRP utilizes carbon fibers for reinforcement, GFRP is composed of glass fibers embedded in a matrix material. Despite the inherent brittleness of both materials, the Young's Modulus of carbon fiber is approximately three times higher than that of glass fiber. Through a comparative analysis of GFRP and CFRP, the influence of various fiber materials on the performance of FRP columns is examined. Models corresponding to Cases 16 through 21 were constructed using the Finite Element Method (FEM) as detailed in Table 2. The parameters for GFRP are sourced from reference^14^, as presented in Table 1. The axial collapse-induced crushing process of the model with different sections in a vertical layup is depicted in Fig. 17. The relationship between load and displacement is illustrated in Fig. 18a. During crushing failure, the load quickly rises to a peak, with the circular section's peak slightly surpassing the hexagonal, both exceeding the square. Subsequently, the load rapidly decreases to a lower stable value and gradually fluctuates.

Upon comparing Figs. 14 and 18, it becomes evident that for both CFRP and GFRP configurations with a [0°]_10_ layup, the load rapidly diminishes to its minimum upon column failure. Failure occurs due to fibers being oriented perpendicular to the cross-section, causing cracks to propagate along the fiber direction, resulting in a distinct “blossoming” shape. Notably, under identical conditions, CFRP exhibits a higher peak load compared to GFRP.

**Figure 19:**
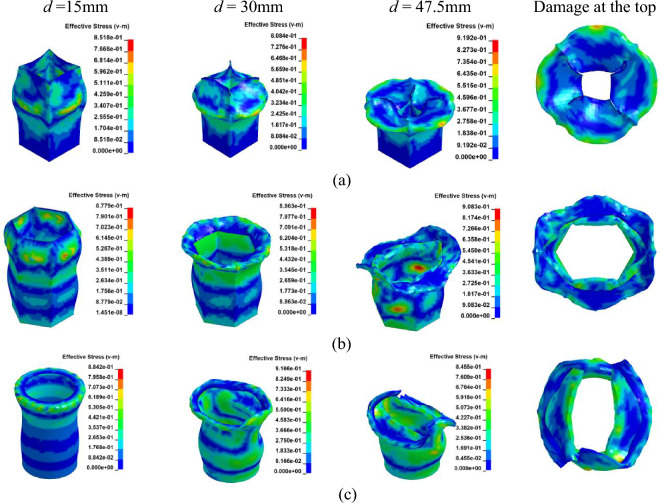
Simulation of the GFRP columns crushing process, at [± 45°]_5_. (**a**) Square section, Case 19. (**b**) Hexagonal section, Case 20. (**c**) Circular section, Case 21.

**Figure 20:**
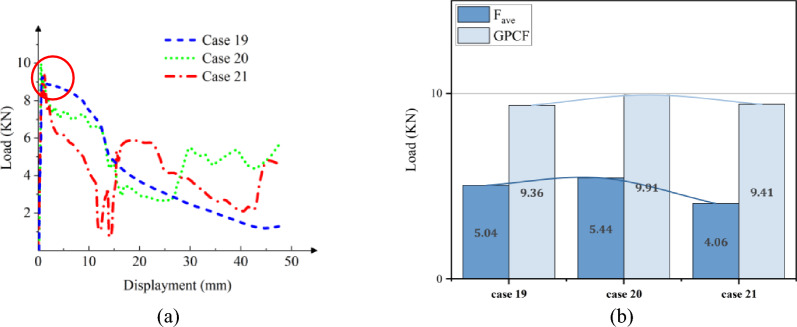
The load–displacement curve of GFRP columns during crushing, at [± 45°]_5_. (**a**) Load and displacement curve for case 19–21. (**b**) Comparison for case 19–21.

Figures 19 and 20a depict the crushing process and load–displacement curve for a [± 45°]_5_ layup, respectively. During compression, the upper column segment undergoes plastic deformation and fracture, displaying weak resistance to vertical loads against the rigid wall. The initial peak load decreases, making it prone to transitioning into the progressive damage energy-absorption phase. Circular cross-sections, compared to other profiles, exhibit more axial fractures, leading to a swift decline in load-bearing capacity and noticeable oscillations in the load curve.

As illustrated in Figs. 18b and 20b, the CFE values for Case 16–18 were 23%, 27%, and 26%, respectively, while those for Case 19–21 were 54%, 55%, and 43%, respectively. The vertical laying of fibers under the same conditions provided higher structural strength, high fluctuation of load force, and lower efficiency of compacting, and when the fibers were laid at 45°, the smaller the fluctuation of load force, and the stable compacting process. In scenarios where fibers are oriented at a 45-degree angle across the cross-section, the column retains a certain degree of structural support even post-damage. Comparing CFRP and GFRP columns, both round and hexagonal cross-sections exhibit minimal differences in performance, as evidenced in Figs. 16b and 20b, with both outperforming the square section. Analysis across various cross-sections reveals that CFRP demonstrates superior load-bearing capacity compared to GFRP, attributable to its higher maximum stress levels within identical cross-sections. Consequently, when budget constraints are not paramount, CFRP emerges as the preferred material for structural columns in the project.

## Conclusions

This article employs the finite element method to simulate the collapse process of fiber-reinforced columns. A comprehensive analysis is conducted on the influence of matrix materials, fibers, cross-sections, and laying angles on columnline mechanical characteristics. The findings are summarized as follows:

(i) In comparison to pure matrix materials, FRP demonstrates enhanced load-bearing capacity and absorption capabilities. Its superior plastic properties, especially when using Al as the matrix material, ensure minimal failures. Consequently, axial column damage results from plastic distortions, leading to layered folds within the column wall. When fragile resin serves as the matrix material, most FRP columns undergo breakage during the crushing process, often displaying a “blossoming” failures pattern. Mechanical performance analysis positions CFRP ahead of resin-based CFRP and Cf/Al configurations.
(ii) The choice of fiber material significantly dictates FRP structural performance. Specifically, CFRP exhibits superior attributes compared to GFRP, particularly when using identical resin types. Within similar cross-sections, CFRP showcases greater resistance to axial forces than GFRP.
(iii) Vertical laying of fibers under the same conditions provided higher structural strength, high fluctuation of load force and lower efficiency of crushing, and when fibers were laid at 45°, the smaller the fluctuation of load force was, and the crushing process was stable. Observations indicate that the principal stress peaks at a 45° angle perpendicular to the shaft during the crushing phase. Consequently, vertical fiber laying demonstrates a higher maximum load than the 45° layup.
(iv) Cross-sectional studies reveal negligible differences in peak load between round and hexagonal columns, both surpassing the square column in performance.

## Data Availability

The datasets produced and analyzed in this study are not publicly accessible at this time. This limitation arises because the research heavily relies on an ongoing major project that remains incomplete. Consequently, the associated research data are not currently available to the public. Interested parties may request access from the corresponding author.
